# Efficacy, safety and cost-effectiveness of a web-based platform delivering the results of a biomarker-based predictive model of biotherapy response for rheumatoid arthritis patients: a protocol for a randomized multicenter single-blind active controlled clinical trial (PREDIRA)

**DOI:** 10.1186/s13063-020-04683-7

**Published:** 2020-08-31

**Authors:** Dalifer Freites-Núñez, Athan Baillet, Luis Rodriguez-Rodriguez, Minh Vu Chuong Nguyen, Isidoro Gonzalez, Jose Luis Pablos, Alejandro Balsa, Monica Vazquez, Philippe Gaudin, Benjamín Fernandez-Gutierrez

**Affiliations:** 1 Rheumatology Department and Health Research Institute, Hospital Clinico San Carlos, Madrid, Spain; 2 Department of Rheumatology, CHU Grenoble, Échirolles, France; 3 Rheumatology Department and Health Research Institute, Hospital Universitario La Princesa, Madrid, Spain; 4 Rheumatology Department and Health Research Institute, Hospital Universitario 12 de Octubre, Madrid, Spain; 5 Rheumatology Department and Health Research Institute, Hospital Universitario La Paz, Madrid, Spain; 6 Rheumatology Department and Health Research Institute, Hospital Universitario Ramon y Cajal, Madrid, Spain

**Keywords:** Rheumatoid arthritis, Personalized medicine, Prediction models, Anti-TNFα agents

## Abstract

**Background:**

Rheumatoid arthritis (RA) is one of the leading chronic inflammatory rheumatism. First-line therapy with synthetic disease-modifying antirheumatic drugs (sDMARD) is insufficiently effective in 40% of cases and these patients are treated with biotherapies. The increased use of these drugs each year is becoming a public health issue with considerable economic burden. This cost is 20 times higher than that of sDMARD. However, among patients treated with biotherapies, clinical practice shows that about one third will not respond to the selected drug. In nonresponse cases, practitioners currently have no choice but to perform an empirical switching between different treatments, because no tool capable of predicting the response or nonresponse to these molecules is currently available.

**Methods:**

The study is a prospective, phase III, controlled, multicenter, and randomized, single-blind (patient) clinical trial, including RA patients with a previous failure to anti-TNF therapies. The main objective is the analysis of the clinical and pharmacoeconomic impact after 6 months of treatment. Intervention arm: prescription of biotherapy (rituximab, adalimumab, abatacept) using SinnoTest® software, a prediction software based on proteomic biomarkers. Control arm: prescription of biotherapy based on current practice, without the SinnoTest® software (any biotherapy). In addition, a substudy will be carried out within this trial to generate a biobank and further analyze the proteomic profile of the patients and their modification throughout the study.

**Discussion:**

This clinical trial study will be the first validation study of a biotherapy response prediction software, bringing personalized medicine into the management of RA. We expect that the findings from this study will bring several benefits for the patient and the Health Care System.

**Trial registration:**

ClincalTrials.gov NCT04147026. Registered on 31 October, 2019.

## Administrative information

**Table:** 

Title {1}	PREDIRA (PRediction mEdical DevIce for Rheumatoid Arthritis): Scale-up of unique predictive online platform highly improving the quality of life of Rheumatoid Arthritis’ patient by personalized and efficient biotherapies prescription
Trial registration {2a and 2b}.	ClinicalTrials.gov, NCT04147026. Registered on 31 October, 2019.
Protocol version {3}	Version N° 1.0 - Date: 20/03/2019
Funding {4}	European Institute of Technology and Innovation (EIT-Health) (#19577)
Author details {5a}	Dalifer Freites Núñez, Luis Rodriguez-Rodriguez, and Benjamín Fernandez-Gutierrez: Rheumatology Department and Health Research Institute, Hospital Clinico San Carlos, Madrid, Spain. Isidoro Gonzalez: Rheumatology Department and Health Research Institute, Hospital Universitario La Princesa, Madrid, Spain. Jose Luis Pablos: Rheumatology Department and Health Research Institute, Hospital Universitario 12 de Octubre, Madrid, Spain. Alejandro Balsa: Rheumatology Department and Health Research Institute, Hospital Universitario La Paz,, Madrid, Spain. Monica Vazquez: Rheumatology Department and Health Research Institute, Hospital Universitario Ramon y Cajal, Madrid, Spain. Athan Baillet, Minh Vu Chuong Nguyen, Philippe Gaudin: Department of Rheumatology, CHU Grenoble, Échirolles, France.
Name and contact information for the trial sponsor {5b}	Fundación para la Investigación Biomédica Del Hospital Clínico San Carlos
Role of sponsor {5c}	Monitoring and management.

## Introduction

### Background and rationale {6a}

Rheumatoid arthritis (RA) accounts for a large proportion of chronic inflammatory rheumatisms (CIR), with a prevalence of 0.4% in the Caucasian population [1] and an incidence of 9/100,000 subjects [2]. Chronic inflammation is clinically manifested by pain and morning stiffness and can lead to joint destruction, often leading to major functional disability in the medium term [3]. RA is the result of the interaction between genetic and environmental factors leading to increased circulating levels of many cytokines. The inhibition of these cytokines, such as tumor necrosis factor (TNFα) or interleukin (IL)-6, is a logical approach, which has introduced profound changes in the management of this condition.

Gradually since the 2000s, biological disease-modifying antirheumatic drugs (bDMARDs) were added to the existing therapeutic arsenal. There are currently available several bDMARDs, including TNFα antagonists, anti-IL-6 receptor, anti-IL-1, a T cell anti-activator, and a CD20+ anti-B lymphocyte [4]. In addition, new bDMARDs are expected to be approved for treatment of RA in the following years, making the therapeutic management of these patients even more complex.

One of the major current challenges in RA management is being able to predict drug responsiveness before treatment initiation [5], as there are no tools able to predict the response to a particular medication [6]. Rheumatologists lack evidence-based knowledge to choose the most suitable DMARD for their patients, and these drugs are prescribed in a “trial-and-error” manner. In case of a lack of response (which has been reported around 30–50% for bDMARDs [3, 7]), the physicians have to perform an empirical rotation between the different drugs, until the correct drug, or combination of drugs for this particular patient is found. Therefore, nonresponders are unnecessarily exposed to undesired adverse events along with the worsening of their physical condition. Furthermore, the ineffective use of bDMARDs has a dramatic economic burden due to their important costs [4]. A study by Meissner and colleagues [8] shows a greater overall economic impact on the health system of patients who changed bDMARD following failure of a first biological compared to those who did not change. This economic burden is even greater when the patient has to change his/her treatment for a second time. This phenomenon seems difficult to reverse in case of initial misdirection, since the probability of a patient who has already received an anti-TNF to respond to another biological treatment decreases gradually according to the increasing number of failures of previous treatments [9]. A tool able to provide a probability score of response or nonresponse to specific treatments could pose an important benefit for clinicians and patients.

In order to improve the response rate to bDMARDs, research for predictive biomarkers of therapeutic response has been active for more than 15 years [10]. Several approaches have been followed to identify biomarkers, including genomics, transcriptomics, epigenetics, and proteomics. However, not all of these strategies have contributed benefits at present. Indeed, in the genomics field [11], the search for candidate genes and the replication of results on different cohorts is fragile and tedious. The hypothesis of several associated genes each having a low impact is preferred over that where few genes each with a significant effect would be involved. Its routine use remains complex. Similarly, epigenetics has provided very preliminary data for the time being [12, 13]. Finally, the study of the transcriptome makes it possible to identify a certain number of genes, but its widespread use by clinicians as part of their regular assessment of their RA patients is difficult on a daily basis [14], and the study of the serum metabolome remains complicated [15].

The proteomic approach, on the other hand, is innovative and simpler to use in routine: it is focused on proteins, which are the terminal elements of cellular actions. It has been emerging in research for about 10 years in rheumatology for the theranostic side [16, 17], but clinical applications are not routine. The latest developments in this area, especially those carried out by members of the PREDIRA consortium, could change the management of these patients [16, 18, 19]. These studies have made it possible to characterize biomarkers differentially expressed at baseline in patients who respond to a bDMARD compared to weak or nonresponders, hoping to optimize a targeted prescription of bDMARDs [16, 17, 20]. In order to allow a rapid translation to clinical practice, our approach prioritizes the selection of biomarkers for which validated diagnostic assays exist, are routinely used, and are commercially available. Several predictive algorithms aimed at 3 classes of bDMARDs have been developed: Anti-TNFa (adalimumab), inhibitors of costimulation of T cells (abatacept), and anti-B-cell antigen CD20:(rituximab).

Based on identified biomarkers and predictive algorithms [16, 18, 19], an application called SinnoTest® has recently been validated [21–23] to determine a priori the most appropriate treatment for the patient with RA according to its clinical context (patients with a previous failure to bDMARDs), depending on its specific proteomic profile (predictive and personalized medicine). This medical device has also been CE marked/approved since August 2018. In practice, from a simple biological sample of a patient, such as blood (plasma or serum), specific protein biomarkers are quantified using routine techniques, allowing after combination through an algorithm, to associate the patient’s profile at a personalized response or nonresponse status for these biotherapy rotational patients. An online interface allows the rheumatologist to access, for each patient, the results of biomarker analysis, as well as the probability of response to different biotherapies available on the market.

We hypothesize that the SinnoTest® software offers real-world clinical and pharmacoeconomic benefits by integrating the main biotherapies currently available. This study will target patients with RA in rotation of a first biotherapy due to inefficiency or toxicity. The expected economic impact is multiple: change in the use of care (hospitalizations, medicines, regular biological controls, outpatient stays, consultations, nursing, surgery, transportation, etc.); reduction of the costs related to the losses of production (reduction of the incapacities at work inducing absenteeism, long-term work stoppages, permanent disability ...); reduction of intangible costs, the assessment of which is difficult but which represents an expected social benefit of such a care strategy. In addition, we will try to show that SinnoTest® also has a strong clinical impact in terms of early-adapted management via the responder rate.

### Objectives {7}

The primary objectives of this clinical trial is to study the clinical and pharmacoeconomic impact after 6 months of the use of the SinnoTest® predictive tool in patients with rheumatoid arthritis who have failed to a first anti-TNF biologic agent compared to usual care. The secondary objectives will be:

- Pharmacoeconomic: to carry out a budget impact analysis (BIA) at 6 and 12 months;
- Clinical: to describe the performance of the software’s predictive model on new clinical data from the 6-month trial.

We will also carry out a proteomics substudy with the objectives of comparing the variation of the proteomic profile between the M0 (date of inclusion) and the M6 (end of study date). The achievement of this objective will be based on the constitution of a biobank, which will serve as a basis for future studies focused on the therapeutic management of these patients.

### Trial design {8}

This project is a prospective phase III randomized clinical trial in 2 parallel groups, multicenter, controlled (prescription of bDMARD with or without the SinnoTest® software), and single-blind (the patient will not know if his bDMARD treatment has been prescribed with or without the help of SinnoTest® software). The inclusion period will be 12 months. Each patient will be followed up to 6 months (clinical evaluation) and up to 12 months (for the analysis of the budgetary impact at 12 months) post randomization.

## Methods: participants, interventions and outcomes

### Study setting {9}

The population studied concerns patients with rheumatoid arthritis, who have failed a first anti-TNF medication (due to inefficiency or adverse events), and attend rheumatology outpatient clinics from tertiary care centers in the Madrid Region (Spain). This situation corresponds to a very large majority of patients admitted to rheumatology consultations.

### Eligibility criteria {10}

Patients meeting the criteria below will be eligible in the study: patients over 18 years old and under 70 years old with RA, defined according to the ACR / EULAR 2010 or ACR 1987 criteria; patients failing a first anti-TNF, defined as ineffectiveness (which is defined as a DAS28-ESR ≥ 3.2 and an inadequate response to iTNF according to the usual rheumatologist, which generally includes one or more of the following conditions: persistent swollen and tender joints, persistence of disease activity according to the overall evaluation of the patient, high levels of acute phase reactants, and/or dependence of analgesics, nonsteroidal anti-inflammatory drugs or corticosteroids), or toxicity (defined as the appearance of any adverse event that the habitual rheumatologist relates to the medication and requires discontinuation); effective contraception for patients of childbearing potential; patients able to read and understand the modalities of the protocol; patients who have dated and signed the informed consent form of the trial; stability of treatments (immunosuppressants, corticosteroids, nonsteroidal anti-inflammatory drugs) between the selection visit and the inclusion visit (M0).

The exclusion criteria will be as follows: Patients who do not meet the criteria below will be eligible in the study; patients with a contraindication to a bDMARD or methotrexate; patients included in another therapeutic evaluation trial during the trial; surgical intervention programmed during the trial; patients with difficulties in understanding the Spanish language; patients who cannot be followed up to 12 months; psychosocial instability incompatible with regular monitoring (homelessness, addictive behavior, antecedent of psychiatric pathology or any other comorbidity that would make it impossible for free and informed consent or limit adherence to the protocol); breastfeeding and pregnancy: although there are bDMARDs that can be used during pregnancy, since SinnoTest may recommend a treatment not recommended for use in pregnancy, it is decided to exclude the recruitment of pregnant subjects.

A previous failure to adalimumab does not constitute an exclusion criterion, and therefore those patients are eligible to participate in this trial.

### Who will take informed consent? {26a}

Informed consent will be obtained by a practicing rheumatologist, able to prescribe any of the drugs that the study subject may receive, and a member of the study team.

The Investigator will be responsible for providing each patient with an information sheet about the trial and the objectives, methods, foreseeable benefits, and potential risks of the study, which should be read by the patient. The investigator must explain to the patients that they are totally free to refuse their participation in the study or to abandon it at any time and for any reason. The Investigator will be responsible for obtaining the written informed consent of each of the participating patients before proceeding with any medical procedure of the study. The investigator will be responsible for not involving any patient in the study without having previously obtained their voluntary consent in writing.

### Additional consent provisions for collection and use of participant data and biological specimens {26b}

In addition, for the proteomics substudy, a second independent informed consent will be collected, which will also include the possibility of storing the samples not used in the present study in the biobank of the center where it is carried out in the study and in the Biobank of the Center Hospitalier Universitaire Grenoble Alpes (France).

### Interventions

#### Explanation for the choice of comparators {6b}

##### Intervention description {11a}

The patients will be randomized to an intervention group (SinnoTest®) or an active-control group (usual care). Patients allocated to the intervention arm will receive the prescription of bDMARD (rituximab, adalimumab, abatacept) using SinnoTest® software, while those allocated to the control arm will receive the prescription of bDMARD without the SinnoTest® software which corresponds to current practice (all bDMARDs).

##### Innovative procedure

*Innovative Medical Device (IMD): SinnoTest® Software version 2.0*

SinnoTest® is a therapeutic guidance device for patients suffering from chronic inflammatory rheumatism, in particular RA. SinnoTest® consists of the following three elements: “routine” biological assay of biomarkers from a blood sample, calculation algorithm, and graphical user interface (software and online application). This device was designed by the company Sinnovial. The assessment of its conformity was carried out by SurgiQual Institute.

Biomarkers are detected and quantified in blood samples of patients, using commercial tests aimed at determining the levels of these molecules. These results are sent to the SinnoTest® secure server. These data are processed by algorithms to determine the probability of response to the treatments available in the therapeutic arsenal (biological therapies). These algorithms were developed in a population of patients with a previous failure to iTNFs receiving the specific medication the algorithm was designed for. This data is sent to a secure server. The graphical user interface is an independent software, accessible through a web platform, which allows authorized health professionals to access the results of the analysis of individual patients. SinnoTest® is a stand-alone IMD-MD software, accessible via a web platform. Rheumatologists from the different centers will be contacted by email to register for the SinnoTest® online platform. A link in this email will provide access to the registration part of the application. The rheumatologists will then be able to connect to the SinnoTest® platform thanks to their personal identifiers.

*Process of the innovative procedure*

The selection of the bio-drug is carried out based on the recommendations of SinnoTest®. This test categorizes bDMARD based on the probability of response. It will allow to prescribe both original molecules and biosimilars in an equivalent way. In the SinnoTest® arm, the investigator prescribes the treatment defined as the most effective by SinnoTest®, except in case of contraindication. If contraindicated, the investigator prescribes the second-choice treatment (if any) of SinnoTest® in terms of efficacy.

The bDMARDs possibly recommended by SinnoTest® are adalimumab, rituximab, and abatacept. Not being exhaustive in terms of biotherapies, it is possible that the SinnoTest® cannot recommend any bDMARD. In this case, the rheumatologist may prescribe one of the following other bDMARDs: etanercept, adalimumab, infliximab, certolizumab, tocilizumab, sarilumab, abatacept, anakinra, golimumab, or rituximab.

##### Reference procedure

There is currently no evidence of treatment response in RA. The choice of the treatment by the rheumatologist is therefore empirical. In order to simplify the process and favoring blinding, regardless of which arm the patient is randomized to, the usual rheumatologist will record two treatment proposals in the clinical history. On the one hand is the treatment that he would prescribe if the patient was randomized to the control arm and, on the other hand, the one that he would prescribe if the patient was randomized to the intervention arm and if SinnoTest® did not recommend any treatment (therefore, the proposed treatment in this case cannot be any of the 3 potentially recommended by SinnoTest®).

At the successive visit after 6–10 days in which the bDMARD will be prescribed, if the patient was randomized to the control arm, or was randomized into an intervention group, but SinnoTest® did not recommend any bDMARD, the medication previously registered by the usual rheumatologist will be used.

*Process of the reference procedure*

This is the current management of patients with RA, based on the EULAR recommendations. In case of rotation of biotherapy after prescription of a first biotherapy, the rheumatologist may prescribe the following bDMARDs: etanercept, adalimumab, infliximab, certolizumab, tocilizumab, abatacept, anakinra, golimumab, rituximab. All these bDMARDS can be prescribed as well as their biosimilars. In case of remission of more than 6 months, a reduction (dosage or spacing of the catch) of the biotherapy can be considered.

##### *Concomitant medications authorized with anti-TNFα, anti-Il-6R, and CTLA-4*

These include any conventional synthetic DMARD that the patient was previously taking before being included in the study and that his rheumatologist deems necessary to continue. Oral corticosteroids at dose ≤15 mg/day, intravenous corticosteroids, intraarticular, and peri-articular local injections of corticosteroids, salicylates, nonsteroidal anti-inflammatory drugs (NSAIDs), analgesics, drugs necessary for the treatment of comorbidities, concomitant treatment with corticosteroids, NSAIDs, and analgesics showed no effect on the pharmacokinetics of these biotherapies.

##### Criteria for discontinuing or modifying allocated interventions {11b}

The discontinuing or modifying allocated interventions for a given trial participant in the study will be as follows: lack of response to medication in the intermediate visit (3 months after inclusion) using the EULAR response criteria; occurrence of adverse events, including malignancy; loss to patient follow-up; withdrawal of consent by the patient; decision of the investigator (inadequate follow-up of the instructions of the doctor or study staff); the rheumatologist or the promoter (medical monitor) decides that continuing the study can be harmful to the patient; in case of pregnancy (only if the patient is under treatment with a therapy that does not recommend pregnancy; in the event that the medication is compatible with the pregnancy, the patient can still be included in the study); in case the patient needs a treatment or has received some treatment not allowed in the study; erroneous inclusion in the study; circumstances not foreseen; cancelation of the study.

Patients discontinuing the intervention will not be followed up and will be withdrawn from the study.

No treatment / procedure prohibited during the study.

##### Strategies to improve adherence to interventions {11c}

No specials provisions will be put in place to improve adherence, besides those already carried out in usual care.

##### Relevant concomitant care permitted or prohibited during the trial {11d}

No restrictions regarding concomitant care were enacted during the trial. The only limitations were those imposed by usual care: concomitant synthetic DMARDs were allowed, corticosteroid dosage could be modified as required, intraarticular corticosteroid injections were allowed.

##### Provisions for post-trial care {30}

All patients will return to standard care after the trial.

### Outcomes {12}

#### Primary outcome measures

##### *Clinical primary outcome measure*

Therapeutic response (time frame: 6 months)

The therapeutic response will be assessed using the EULAR response criteria [24] at 6 months, which is defined by a low disease activity (DAS28-ESR < 3.2) and a decrease in the DAS28-ESR > 1.2 points from baseline (further details regarding disease activity in the “Clinical secondary outcome measures” section).

#### Secondary outcome measures

##### *Pharmacoeconomic secondary outcome measures*

Incremental cost utility ratio at 6 months (time frame: 6 months)

This outcome will be calculated as the average differential cost per patient between both study arms (mean costs of the SinnoTest® arm − mean costs of the control arm) divided by the difference in effectiveness between both study arms measured in the number of years of life weighted by the quality of life (QALY: quality-adjusted life year) generated by each of the strategies (mean QALY of the SinnoTest® arm − mean QALY of the Control Arm).

QALY will be measured using the EuroQol-5D-5L. The Spanish value set will be used to obtain the EQ-5D index values. Cost will be considered from a Societal perspective, including both direct and indirect costs The ratio will be expressed in cost (2019 Euros) per QALY earned, which represents the additional cost that will have to be spent to earn a healthy year of life.

As direct costs, we will consider concomitant medications, procedures, outpatient consultations (both primary and specialized care), hospitalizations, and complementary test (laboratory, radiology…). Regarding indirect costs, we will consider sickness absences, transportation, home assistance (both by professional and non-professional caregivers), and home modifications related to the disease.

Regarding the determination of unit costs, we will favor, where possible, a cost-of-production approach:

- The prices of drugs dispensable through pharmacies will be obtained from the Database of the Spanish Official College of Pharmacists
- The prices of hospital dispensing drugs will be obtained from the Pharmacy Services of each participating center.
- The costs of the different medical acts (first visits or revisions, in primary or specialized care, by different medical professionals) will be provided by each participating center.
- The costs of hospitalizations and the procedures that are carried out will be provided by each participating center.
- The costs of the complementary tests will be obtained from each participating center.
- The use of home assistance will be evaluated based on a standard average cost. The cost of employing professionals from the medico-social sector (for example, occupational therapist) will be estimated based on an average cost per hour.
- The cost of improvements in the home will be estimated, as far as possible, in the billing data or in the data obtained by the professionals of the sector involved.
- In relation to the costs of caregivers (non-professionals) and the loss of production of the patient, according to the recommendations, the direct costs will take into account the time of the people intervened and the time that the caregivers dedicate to the care of the patients. However, in relation to the valuation method, there is no agreement on the method to be used. We will favor the human capital method, where the “value” of an individual is estimated by its productive value in the labor market, that is, through the gross salary. These productive costs will be obtained through the data of age, sex, and professional sector of the individual through the national statistics available on the Spanish Social Security website.
- The cost of medical transport will be assessed through the rates established based on the distance and type of vehicle.

We consider that 6 months is enough time to assess the performance of the intervention. According to the EULAR recommendations for biotherapy management in rheumatoid arthritis [25], 6 months is the time to assess if the therapeutic objectives of the medication has been achieved (remission or at least low disease activity). Based on those recommendations, if the patient is still active, treatment should be modified.

Incremental cost-effectiveness ratio at 6 months (time frame: 6 months)

This outcome will be calculated as the average differential cost per patient between both study arms (mean costs of the SinnoTest® arm − mean costs of the control arm) divided by the difference in effectiveness between both study arms measured as the percentage of patients achieving a good clinical response in each study arm (% in the SinnoTest® arm − % in the control arm).

Good clinical response will be measured using the EULAR criteria of good clinical response. Cost will be considered from a Societal perspective, including both direct and indirect costs. The ratio will be expressed in cost (2019 Euros) per increase in 1% of subjects achieving a good clinical response, which represents the additional cost that will have to be spent to earn a healthy year of life rates of treatment response patients associated respectively with the usual strategy without SinnoTest® and with the strategy with SinnoTest®.

Budget impact analysis at 6 and 12 months (time frame: 12 months)

A budget impact analysis will be carried out if the innovation is deemed efficient, meaning, if the main outcome is achieved (SinnoTest is cost-effective). A 12-month horizon was chosen to make sure that the differences between both study arms were not compensated in the long term.

This budget impact analysis will describe the resources consumed and the expenses generated by each scenario, a scenario with the use of SinnoTest® and a scenario without SinnoTest®. The same direct costs and the same method of reporting will be used as those used in the main outcome assessment.

##### *Clinical secondary outcome measures*

Disease activity, quality of life, and disability (time frame: 6 months)

RA disease activity will be evaluated using both the DAS28-ESR and CRP indexes. They are composite scores derived from 4 measurements: number of swollen joints (out of 28), number of painful joints (out of 28), global assessment by the patient of RA activity on a scale visual analog of 0 to 10 cm (0 = no manifestation of RA, 10 = maximum severity that the patient can imagine), and either erythrocyte sedimentation rate (ESR, in mm/h) OR C-reactive protein (in mg/dL). Quality of life will be measured with the EQ5D-5 L. Disability will be measured using the Health Assessment Questionnaire (HAQ).

Software’s predictive model performance (Time Frame: 6 months)

Sensitivity, specificity, and positive and negative predicted values of the predictive models using the biomarkers will be assessed on the new clinical data from the 6-month trial.

##### *Proteomic secondary outcome measures*

Description of the variation of the proteomic profile between M0 (biotherapy start date) and M6 (6 months visit) (time frame: inclusion and 6 months)

Based on shotgun and semi-quantitative proteomics, the differences between the proteomic profile at baseline and at M6 will be analyzed.

### Participant timeline {13}

This study will be conducted by rheumatologists who have the opportunity to follow patients with RA and to conduct this study in good conditions and in accordance with regulatory and legal recommendations. The only difference in follow-up between the 2 groups is the addition of the “SinnoTest® Protein Biomarker” assay and the use of the results of this assay for therapeutic management.

Patients will be seen as part of their follow-up consultation in the Rheumatology department:

-Screening: verification of the eligibility criteria.
-Inclusion or M0 visit: after information and signature of informed consent. All patients will have a SinnoTest® blood sample. At the end of this visit, the patient is randomized to the SinnoTest® group or the control group. In the control group, the SinnoTest® sample is kept but not analyzed.
-6–10 days after the consultation, the prescription of the biotherapy recommended by the rheumatologist is sent by post. This prescription uses SinnoTest® results in the SinnoTest® arm.
-Followed quarterly: M3 and M6.

As part of the proteomics substudy, for those patients who agree to participate, blood samples will be collected from the 2 groups in the M0 and in the M6 to study the variations in the proteomic profile of the patients. In addition, the remaining samples will be deposited in a biobank (in the recruitment center and in the Biobank of the Center Hospitalier Universitaire Grenoble Alpes, France). The participant timeline is presented in Table 1.

**Table 1:** Schedule of enrollment, interventions, and assessments

Visits	Selection	Inclusion^a^ M0	M3 ± 2 weeks	M6 ± 2 weeks	M12 ± 2 weeks
Information^a^	✓				
Information and consent^b^		✓			
Confirmation of eligibility		✓			
Clinical/physical examination, rheumatoid arthritis		✓	✓	✓	
Routine blood analysis		✓	✓	✓	
Questionnaire EQ5D		✓	✓	✓	
Questionnaire HAQ		✓	✓	✓	
Biomarkers^c^		✓			
Patient booklet^d^		✓	✓	✓	✓
Administration/dispensation of biotherapy^e^		✓			
Biobank^f^		✓		✓	
Concomitant treatments		Collection throughout the study			
Adverse effects collection		Collection throughout the study			

^a^ Inclusion: The inclusion visit should be done in 2–3 weeks

^b^ Information and informed consent: The patient will sign informed consent for the main study and additional consent (optional) if they accept 6-month and 1-year inclusion samples, which will be retained for the creation of biobank

^c^ Biomarkers: A blood sample will be taken for the determination of SinnoTest® biomarkers in the intervention arm patients

^d^ Patient booklet: The rheumatologist or CRA investigator will deliver the booklet to the patient at the inclusion visit. The patient brings the notebook during each follow-up visit and will carry it with him after the visit. At the end of the follow-up, he will return his completed patient booklet to the CRA / TEC of the Investigator Center by mail using the envelope provided with the notebook

^e^ Administration/dispensation of biotherapy: Patients in the control group will receive their biotherapy prescription in current practice. For patients in the intervention group, the rheumatologist will prescribe the biotherapy selected by SinnoTest®. The results of the pregnancy test should be negative

^f^ Biobank: A blood test will be carried out in all included patients who signed the additional consent for the creation of a biobank

### Sample size {14}

To re-evaluate the metrological properties of SinnoTest®, including 90 patients per group will highlight a differential response rate between the software arm and the control arm of more than 18.2%, considering a current response rate of 65% (defined as an EULAR good response [24]) with conventional care (control arm). The number of subjects required was estimated considering a bilateral alpha risk at 5% and a power of 80%. In total, 180 patients will be the estimated number of participants needed to achieve study objectives.

Due to the subject’s and the study’s characteristics, no allowance for loss to follow-up was considered in the sample size calculation: patients with a chronic disease, probably being followed up in the same center and by the same rheumatologist for some time (> 1 year), and the short follow-up time for the primary outcome.

### Recruitment {15}

Recruitment will be done among patients coming for rheumatology consultations in the 5 centers participating in the study. Potentially eligible patients will be identified in the everyday clinical practice of the research staff, or referred to them for assessment of eligibility having been identified by rheumatologists who are not research staff. Medical records will be checked to identify any other potentially eligible patients. The patient’s eligibility will be confirmed by the responsible researcher. After confirmation, any patient who agrees to participate in the research must sign the informed consent to begin participation.

### Assignment of interventions: allocation

#### Sequence generation {16a}

It was centrally generated at the Hospital Clínico San Carlos Clinical Research Unit.

#### Concealment mechanism {16b}

Only the person that has generated the list has access to it.

#### Implementation {16c}

The eligible patient will be randomized, with a 1:1 allocation, via an internet server (access by secure code 24/24; RedCap) in one of two groups: SinnoTest group or control group. A blood sample will be extracted from the patient randomized in the software arm for the determination of selected biomarkers for the use of SinnoTest®. A patient randomized to the control arm will also have his/her blood drawn (but this one will not be analyzed) so that the two arms are identical and to maintain blinding for the patient.

The coordinating center will manage its development and its availability on the internet. It will only be done after informing the patient and signing the consent. The randomization will be individual with blocks of random size, stratified by center. The investigator or CRA will connect to the server after confirming that the patient meets all the inclusion/exclusion criteria. Block randomization will ensure that an equal number of patients are randomized to each study arm and to each study site.

The involvement of 5 centers in this study allows individual randomization without fear of the existence of a contamination bias. Indeed, investigators may change their empirical management under study because of patient outcomes previously included with SinnoTest®, but the relatively low inclusion counts per center suggest that this bias will be negligible. In addition, in the control arm, the treatments will be prescribed by the rheumatologist who usually treats the patient, so the number of patients who will have the opportunity to “learn” is even lower, because it is shared among all doctors that include patients in the study.

This design also neutralizes the disappointment bias of investigators who can all use the innovation under study. By stratifying by center, about half the patients will have their treatment prescribed by SinnoTest, so they can be sure they will have the opportunity to use the software and assess its functions.

### Assignment of interventions: blinding

#### Who will be blinded {17a}

Study subjects will be blinded to the intervention (who decides the medication prescribed). Evaluators will also be blinded to the intervention where the subject was allocated.

#### Procedure for unblinding if needed {17b}

This is a single-blind study (single blind). Only the doctor knows the details of how to prescribe treatment (i.e., the doctor knows if the patient is in the control group or the group using the SinnoTest® software). Lifting of the blind is not applicable.

The investigator carrying out the aleatorization will be responsible for the blinding of the subject.

### Data collection and management

#### Plans for assessment and collection of outcomes {18a}

The use of the SinnoTest® V1 device is reserved for hospital rheumatologists. Training in the use of the graphical interface will be dispensed. In addition, manuals have been created for the use of the device and for the collection, processing, and delivery of samples.

#### Plans to promote participant retention and complete follow-up {18b}

In order to maximize the retention rate of the subjects included in the study, several strategies will be put in place:

- Participants will be asked to provide both phone and email addresses, as well as the best time to contact them.
- Study subjects will also be educated in the significance of research follow-up even if they decide to discontinue the study drugs. Explaining how their experiences with the treatments, whether positive or negative, are critical to evaluating the intervention under study can increase their understanding of the research process and lay the groundwork for future discussions about the importance of completing research assessments.
- Anticipated barriers to attending appointments such as transportation, work schedules, vacations, and childcare can be reviewed up front and resolved.
- Phone calls regarding study visit reminders will be used for all appointments, and rapid follow-up of missed appointments will be incorporated into the protocol.
- The schedule of study visits will be flexible, ensuring subject retention.

### Data management {19}

Electronics case report forms (eCRFs) will be used that comply with the general and specific good practice standards, as well as the highest requirements for computer validation, with restricted access at the user level, provided with inconsistency detection filters and with traceability of all the information until the final closing of the study. CRFs must be completed for each subject screened/enrolled in this study. This also applies to patients who do not complete the full follow-up planned in the trial.

All CRFs must be filled out by the personnel duly authorized to do so, who will have access codes to the application for entering personal and non-transferable data. The investigator will keep the records and data during the trial in compliance with all legal and regulatory provisions in force. All data must be supported by original documents in the test center. Any record or document used as a source of information (which will be called the “original data of the subject”) will be kept for review by authorized representatives of the promoter or regulatory bodies. The CRFs will be filled in as soon as possible after the evaluation has been carried out.

All the dates that appear in the CRFs referring to analytical tests and other data must coincide with the dates in which the samples were obtained or the procedures were performed.

#### Study database

To facilitate the statistical analysis, a computerized database will be created in which the integrity of the data from the CRFs will be recorded, so that an exact replica of the information contained in them is created.

A data management plan will be made before the beginning of the definition of the database in which the recording process and the errors and consistency controls that will be performed on the recorded data will be detailed. A dictionary of variables will be generated in which the correspondence between the data contained in the CRFs and the variables of the database will be detailed, as well as the codifications used and the meaning of the recorded values.

In the event of inconsistencies or errors in the data, requests for clarification will be generated for the researchers for their verification or correction, which will be treated in an equivalent way to the CRFs. Access to the database will be restricted to the Data Manager (design, input, and data cleansing) and the personnel in charge of data transcription (data entry).

Prior to the declaration of the definitive database, a verification is done of the consistency of the values of the inclusion/exclusion criteria, of the clinical evaluations, of the results of complementary explorations, of the dates of the visit, of the compliance, of the medication received, of the adverse events, of the information about dropouts, and of the evaluation of effectiveness.

A definitive database will be declared that will be registered with signature and date. Two protected copies of the same will be kept, and paper lists of the variables contained in the database will be generated for archiving. The final database will be used for statistical analysis.

#### Registry and file maintenance

All the essential documentation of the clinical trial will be filed in a master file of the study, whose safe and complete conservation will be ensured for the time required according to the legislation in force and at the disposal of the authority that requests it. This documentation will include the following: work protocol (final version) and amendments; models of all the employed versions of information sheet and informed consent form; CREC permits; authorizations of the health authorities; curriculum vitae of all the personnel participating in the study; random assignment list and treatment allocation codes; individual data collection notebooks; documentation related to the study monitoring procedures; documentation of the study database and definitive database; documentation of data management and clarification requests; statistical analysis; adverse event notifications; final report; certificates of audits; standardized work procedures applied in the study; study financing and payments; correspondence.

### Confidentiality {27}

The investigator will ensure the right to privacy of patients and must protect their identity against unauthorized third parties. The study monitor may have access to the patient’s identity and data in relation to the study’s monitoring procedures.

The investigator will keep a patient identification list updated with the correspondence between the name, clinical history number, and the patient’s identification number or code for the clinical trial, which will be kept together with the patients’ informed consent forms in a file unique in the center. The full name of the patient should not appear in any other section of the data collection notebooks or study documentation. At the end of the study, a copy of the list in which the names of the patients will be hidden will be included in the file of the researcher of the study.

In case that an audit of the study is conducted, the auditors who perform it, as well as the health authorities that may require it for regulatory purposes related to the study, may also have access to patient data.

All participants in this research project expressly commit themselves not to disclose the identity of the treated patients and to respect the rules of confidentiality regarding the data and information to which they have access when participating in the trial. The personal data collected and stored for the purpose of this study will be treated in accordance with the provisions of the General Data Protection Regulation (GPDR: Regulation EU 2016/679).

### Plans for collection, laboratory evaluation, and storage of biological specimens for genetic or molecular analysis in this trial/future use {33}

In the case a subject agrees to participate in the proteomics substudy, after carrying out the planned experiments during the trial, the remaining biological samples will be donated to the biobanks belonging to the corresponding ISCIII Biobank Network of each participating center and the Biobank of the Center Hospitalier Universitaire Grenoble Alpes (France). These samples may be transferred to other researchers, in accordance with current regulations (Biomedical Research Law 14/2007 and Royal Decree 1716/2011), to carry out studies related to their disease. Any project for which samples are used will be previously approved by an accredited Ethics Committee.

### Statistical methods

#### Statistical methods for primary and secondary outcomes {20a}

##### Description of the population studied

The study population will be described by a flow diagram according to the CONSORT recommendations (CONsolidated Standards Of Reporting Trials). The different variables (demographic and biological) will be described by treatment arm: for quantitative variables, number of data analyzed, mean, and standard deviation, Q1, Q3, minimum, maximum, and median, and for qualitative variables, number of analyzed data, absolute frequencies, and percentage.

#### Primary outcome measures

##### *Clinical primary outcome measures*

Therapeutic response

Therapeutic response at 6 months will be described using number of analyzed data, absolute frequencies, and percentage.

Comparison between the study arms will be performed using logistic regression models. Models will be adjusted by baseline variables, and they will allow us to estimate an effect size for the difference between study arms regarding the clinical variables.

#### Secondary outcome measures

##### *Clinical secondary outcome measures*

Disease activity, quality of life and disability

Clinical parameters at 6 months will be described using number of analyzed data, mean, standard deviation, Q1, Q3, minimum, maximum, and median, for DAS28-ESR, DAS28-CRP, EQ5D-5L, and HAQ.

Comparison between the study arms will be performed using linear regression models. All models will be adjusted by baseline variables, which will allow us to estimate an effect size for the difference between study arms regarding the clinical variables.

In addition, as disease activity, quality of life, and disability will be assessed at baseline, 3 months, and 6 months, their evolution will be analyzed using generalized estimating equations models nested by patient [26, 27] and adjusted by study visit and study arm, using a Gaussian family and Identity as link function. Different covariable structures will be tested (independent and exchangeable) and compared using the Quasi-Akaike Information Criterion [28]. Time × study arm interactions will assess different effects of time in the evolution of DAS28-ESR, DAS28-CRP, EQ5D-5L, and HAQ by study arm. A *p* value < 0.05 will be considered as a significant interaction. For each outcome, in case the interaction is not significant, we will consider that there is no statistically significant difference in the evolution of that outcome between study arms.

Software’s predictive model performance

Sensitivity, specificity, and positive and negative predictive values, and likelihood ratio will be described. The performance of the SinnoTest® will be calculated in the “SinnoTest®” arm by comparing the response predicted by the software and the response observed at 6 months. It will be analyzed globally and for each of the biotherapies.

##### *Pharmacoeconomic secondary outcome measures*

Incremental cost utility ratio at 6 months

The statistical analysis will proceed as follows:

1. Calculating the 6-month incremental cost-utility ratio from the community perspective using the following parameters: difference of the averaged total cost per patient and difference in the average number of QALYs generated by the strategies on both study arms. The ratio will be expressed in cost per QALY earned, which represents the additional cost that will have to be spent to earn a healthy year of life.
  QALYs will be calculated from the answers provided by EQ5D-5L, based on a weighting system reflecting preferences on the health status of the general Spanish population (41). The EQ5D questionnaire will be filled in by the patient at the inclusion visit and then at M3 and M6. The QALYs will be calculated by the area under the curve assuming a linear evolution of the quality of life between the measurement times. The average number of QALYs will be calculated for each of the strategies under study.
  In case of missing data, multiple imputation techniques will be carried out, in addition to sensitivity analysis, with the objective of checking that the assumptions made in the imputation were correct, and checking whether the conclusions of the study are modified, or not, according to the analysis strategy adopted.
  Although subjects will be followed up to 12 months, ICER will only be calculated at 6 months: as pointed out, according to the EULAR recommendations for biotherapy management in rheumatoid arthritis [25], 6 months is the time to assess if the therapeutic objectives of the medication has been achieved (remission or at least low disease activity). Based on those recommendations, if the patient is still active, treatment should be modified.
2. Sensitivity analysis: Deterministic sensitivity analysis: it allows to take into account the uncertainty on certain parameters that can influence the cost of the strategies. We will consider the best and worst scenarios. Taking into account the variability in direct cost associated with treatment in RA [29], we will consider a ± 20% cost increase/decrease. This analysis will take into account in particular the impact of the cost of SinnoTest®, the impact of a change in the cost of treatments through biotherapy, the impact of the cost of work stoppages and the time of the caregivers, and probabilistic sensitivity analysis: it will include the calculation of the confidence intervals of the cost-utility ratio by the nonparametric Bootstrap method and the plot of the associated acceptability curve.
3. In addition to the cost-utility ratio, differences in cost and utility will be analyzed separately: Comparison of the cost of the two strategies: the difference in costs between the groups will be tested by a Student test or a nonparametric test (Mann-Whitney tests) in case of non-Gaussian distribution. The choice of the test will be made with regard to the distribution of costs in each group (Shapiro-Wilk test, on raw data or, if necessary, on transformed data. In addition, distribution will also be assessed using histograms). The construction of the average cost confidence interval will be based on the nonparametric Bootstrap method and comparison of the utility: The number of QALYs will be calculated taking into account the time elapsed between two successive measurements. If the initial utility level between the groups differs (> 10%), a comparison of the number of QALYs between the groups will be made from a linear model to adjust for this level of utility [30]. Given the time horizon, no updates will be made; those of the inclusion visit (M0) will be taken.
4. Multivariable linear regression method will be used to determine the explanatory factors of the average cost per patient of the strategies (i.e., age, sex, socio-professional category). In a first step, bivariable analysis will be carried out. Those variables with a *p* value < 0.15, plus age and gender, will be included in the multivariable analysis. Different models will be compared using the Akaike Information Criteria.

##### Incremental cost-effectiveness ratio at 6 months

The incremental cost-effectiveness ratio at 6 months will be calculated from the cost differential of the strategies and the efficiency differential defined by the rate of good responder patients in each group. The ICER and the 6 months will be subjected to a deterministic and probabilistic sensitivity analysis.

##### *Protomic secondary outcome measures*

Description of the variation of the proteomic profile

The biomarkers of the proteomic profile will be compared between the inclusion visit (M0) and the 6-month visit, as well as the differences in both the M0 and M6 visits between the two treatment arms.

The comparison will be done by biomarker. The biomarkers will be described at each time (indicators and boxplot) globally, as well as by the observed response group. The risk of the first species *α* is fixed, by convention, at 5% for the different comparative analyses.

### Interim analyses {21b}

No formal interim analysis will be carried out.

### Methods for additional analyses (e.g., subgroup analyses) {20b}

The influence of factors on the average cost per patient will also be analyzed by treatment arm. To carry out this analysis, multivariable linear regressions models will be developed and an interaction between demographic and clinical-related variables and study arm will be introduced to assess a significant difference between the association between factor and average costs by study arm.

### Methods in analysis to handle protocol non-adherence and any statistical methods to handle missing data {20c}

Analyses will be carried out by intention to treat (i.e., each patient will be considered to belong to the group to which they were randomized, regardless of the intervention they receive).

For each patient that leaves the study before its completion, the cause will be collected, which will be classified as follows: lack of response to biotherapy, occurrence of adverse events, loss to follow-up, withdrawal of consent, the investigator’s decision, use or need for medication not allowed, erroneous inclusion in the study, unforeseen circumstances, or cancelation of the study.

To handle missing data, multiple imputation techniques will be applied [31], in addition to sensitivity analysis with the object to check the assumptions made in the imputation, and check whether the conclusions of the study are modified, or not, according to the analysis strategy adopted. The multiple imputed dataset will be used to perform the primary and secondary outcome analyses.

In addition, we also will carry out a per-protocol analysis of the primary and secondary outcomes.

### Plans to give access to the full protocol, participant-level data, and statistical code {31c}

A summary of the final version of the study protocol will be made available through the Project’s website (https:\crpredira.eu).

Regarding the access to participant-level data, the promoter will be the only one with access to the participant-level data, following the regulation on data protection. The promoter can share the data with other researchers upon request and within the legal situation.

### Oversight and monitoring

#### Composition of the coordinating center and trial steering committee {5d}

There was no independent oversight committee that oversaw the trial conduct and patient safety throughout the recruitment and follow-up. Considering that we are using bDMARDs which are currently being prescribed in patients with similar characteristics to those included in the study, with a known security profile, we did not consider that the patients are being exposed to a higher adverse event risk compared with the standard clinical practice

#### Composition of the data monitoring committee, its role and reporting structure {21a}

Not applicable.

### Adverse event reporting and harms {22}

In each study visit, the presence of the most common adverse events related to the study medication will be actively inquired by the blind investigator, including

-Infections including pulmonary and urinary infections and opportunistic infections including tuberculosis;
- Nausea, vomiting, abdominal pain, diarrhea, tiredness, dizziness, and headache;
- Liver problems;
- Hematological disorders: agranulocytosis, thrombocytopenia, anemia, aplasia;
- Demyelinating diseases: multiple sclerosis and optic neuropathies;
- Heart failure;
- Stevens-Johnson syndromes and Lyell syndromes;
- Psoriasis;
- Interstitial pneumonitis;
- Hypersensitivity reactions: fever, chills, pruritus, urticarial, dyspnea, chest pain, hypotension or arterial hypertension, and reactions to injection sites

Regarding the notification of severe adverse events, we will follow the definitions and guidelines of the document MEDDEV 2.7/3 review 05/03/2015 “Guidelines on medical devices: Clinical investigations: Serious adverse event reporting under Directives 90/385/EEC and 93/42/EEC”.

All adverse events will be followed until its resolution, subject’s death, or lost to follow-up.

### Frequency and plans for auditing trial conduct {23}

There is a monitoring plan in place, both to assess data completeness and subjects’ safety. This plan includes online/face-to-face monitoring visits every five recruited subjects.

### Plans for communicating important protocol amendments to relevant parties (e.g., trial participants, ethical committees) {25}

The principal investigator (PI) will notify the sponsor of this project of any changes to the protocol and will submit the changes for review to the local CREC. The PI will notify the clinical research center (study site) of any protocol changes and will update the protocol in the clinical trial registry.

### Dissemination plans {31a}

During and after the study, the ClinicalTrials.gov database will be replete with data. After completing the research and processing the data, an article will be written for later publication in an international journal for the dissemination of data and results.

In addition, the study subjects will be informed of the trial results.

## Discussion

This single-blind controlled multicenter clinical trial study is the first validation study of a bDMARD response prediction software, bringing personalized medicine into the management of inflammatory rheumatism. This interest is also shared by pharmaceutical companies who wish to improve the stratification of patients and thus offer more targeted treatments.

We believe that the findings from this study will bring several benefits for the patient and for the health system as reducing patient exposure to ineffective and potentially poorly tolerated bDMARDs controls RA activity. The SinnoTest® will maximize the chances of successful treatment with biotherapy, the patient will benefit from the most appropriate treatment at the right time, which will improve its quality of life. In addition, it is important to consider the high proportion of patients receiving in clinical practice the bDMARDs which can be suggested by the SinnoTest® (up to 49% of the RA patients receiving a bDMARD [32]).

For public health, the short-term benefit will focus on optimizing the management of RA, improving and adjusting the SinnoTest® predictive software, thanks to the clinical results obtained. Data from clinical validation studies will be used to validate and improve the existing algorithm for performance, ergonomics, and functionality and anticipate access to the market by collecting medico-economic data and pursuing the search for partners to allow rapid deployment of the test in clinical practice. The SinnoTest® will allow the physician to optimize the selection of the biotherapy in real time.

The combination of SinnoTest® algorithms will optimize the selection of biotherapy for RA patients, and given the minimal risk for patients and the expected collective benefit, the benefit-risk ratio is very favorable.

## Trial status

The study is in the data collection phase. Recruitment started in December 2019 and was halted in March 19, 2020, due to the SARS-CoV-2 pandemic. On June the 1, 2020, recruitment was restarted and it is estimated to end in January 2021. The current protocol is version 1.0, created in March 2019 and approved by the Clinical Research Ethics Committees (CREC) of the participating centers, as well as the Spanish Agency of Medicines and Health Products in December 2019 before starting the study. This study is registered at Clinicaltrials.gov, identifier: NCT04147026. Registered on 31 October, 2019.

## Data Availability

Not applicable.

## Abbreviations

RA: Rheumatoid arthritis
CIR: Chronic inflammatory rheumatism
sDMARD: Synthetic disease-modifying antirheumatic drug
TNFα: Tumor necrosis factor
bDMARDs: Biological disease-modifying antirheumatic drug
tsDMARDs: Targeted synthetic disease-modifying antirheumatic drug
EULAR: The European League against Rheumatism
HCRP: The Hospital Clinical Research Program
AS: Ankylosing spondylitis
QALY: Quality-adjusted life year
IMD: Innovative Medical Device
eCRFs: Electronics case report forms
CREC: Clinical Research Ethics Committees
PI: Principal investigator
GDPR: General Data Protection Regulation
CONSORT: CONsolidated Standards Of Reporting Trials

## References

[1] Guillemin F, Saraux A, Guggenbuhl P, Roux CH, Fardellone P, Le Bihan E, et al. Prevalence of rheumatoid arthritis in France: 2001. Ann. Rheum. Dis. 2005;64:1427–30. 10.1136/ard.2004.029199 PMC1755224 15800010

[2] Smolen JS, Aletaha D, McInnes IB. Rheumatoid arthritis. Lancet. 2016;338:2023–38. 10.1016/S0140-6736(16)30173-8 27156434

[3] Maillefert JF, Combe B, Goupille P, Cantagrel A, Dougados M (2003). Long term structural effects of combination therapy in patients with early rheumatoid arthritis: five year follow up of a prospective double blind controlled study. Ann Rheum Dis.

[4] Kievit W, Van Herwaarden N, Van Den Hoogen FH, Van Vollenhoven RF, Bijlsma JW, Van Den Bemt BJ (2016). Disease activity-guided dose optimisation of adalimumab and etanercept is a cost-effective strategy compared with non-tapering tight control rheumatoid arthritis care: analyses of the DRESS study. Ann Rheum Dis.

[5] Smolen JS, Aletaha D. Rheumatoid arthritis therapy reappraisal: Strategies, opportunities and challenges. Nat. Rev. Rheumatol. 2015;11:276–89. 10.1038/nrrheum.2015.8 25687177

[6] Fleischmann R, Connolly SE, Maldonado MA, Schiff M (2016). Brief report: estimating disease activity using multi-biomarker disease activity scores in rheumatoid arthritis patients treated with abatacept or adalimumab. Arthritis Rheumatol.

[7] Combe B (2004). Should patients with recent-onset polyarthritis receive aggressive treatment?. Jt Bone Spine.

[8] Meissner B, Trivedi D, You M, Rosenblatt L (2014). Switching of biologic disease modifying anti-rheumatic drugs in patients with rheumatoid arthritis in a real world setting. J Med Econ.

[9] Rendas-Baum R, Wallenstein GV, Koncz T, Kosinski M, Yang M, Bradley J (2011). Evaluating the efficacy of sequential biologic therapies for rheumatoid arthritis patients with an inadequate response to tumor necrosis factor-α inhibitors. Arthritis Res Ther.

[10] Park Y-J, Chung MK, Hwang D, Kim W-U (2015). Proteomics in rheumatoid arthritis research. Immune Netw.

[11] Prajapati R, Plant D, Barton A. Genetic and genomic predictors of anti-TNF response. Pharmacogenomics. 2011;12:1571–85. 10.2217/pgs.11.114 22044414

[12] Duroux-Richard I, Pers YM, Fabre S, Ammari M, Baeten D, Cartron G (2014). Circulating miRNA-125b is a potential biomarker predicting response to rituximab in rheumatoid arthritis. Mediat Inflamm.

[13] Krintel SB, Dehlendorff C, Hetland ML, HØrslev-Petersen K, Andersen KK, Junker P, et al. (2016). Prediction of treatment response to adalimumab: a double-blind placebo-controlled study of circulating microRNA in patients with early rheumatoid arthritis. Pharmacogenomics J.

[14] Smith SL, Plant D, Eyre S, Barton A. The potential use of expression profiling: Implications for predicting treatment response in rheumatoid arthritis. Ann. Rheum. Dis. 2013;72:1118–24. 10.1136/annrheumdis-2012-202743 23486412

[15] Tatar Z, Migne C, Petera M, Gaudin P, Lequerre T, Marotte H (2016). Variations in the metabolome in response to disease activity of rheumatoid arthritis. BMC Musculoskelet Disord.

[16] Trocmé C, Marotte H, Baillet A, Pallot-Prades B, Garin J, Grange L (2009). Apolipoprotein A-I and platelet factor 4 are biomarkers for infliximab response in rheumatoid arthritis. Ann Rheum Dis.

[17] Obry A, Hardouin J, Lequerré T, Jarnier F, Boyer O, Fardellone P (2015). Identification of 7 proteins in sera of RA patients with potential to predict ETA/MTX treatment response. Theranostics..

[18] Baillet A. Protéines S100A8, S100A9 et S100A12 : marqueurs inflammatoires ou acteurs physiopathologiques de la polyarthrite rhumatoïde. Rev. Med. Interne. 2010;31:458–61. 10.1016/j.revmed.2009.10.435 20398973

[19] Baillet A, Trocmé C, Berthier S, Arlotto M, Grange L, Chenau J (2010). Synovial fluid proteomic fingerprint: S100A8, S100A9 and S100A12 proteins discriminate rheumatoid arthritis from other inflammatory joint diseases. Rheumatology (Oxford).

[20] Nair SC, Welsing PMJ, Choi IYK, Roth J, Holzinger D, Bijlsma JWJ (2016). A personalized approach to biological therapy using prediction of clinical response based on MRP8/14 serum complex levels in rheumatoid arthritis patients. Kuwana M, editor. PLoS One.

[21] Nguyen MVC, Baillet A, Romand X, Trocmé C, Courtier A, Marotte H (2019). Prealbumin, platelet factor 4 and S100A12 combination at baseline predicts good response to TNF alpha inhibitors in rheumatoid arthritis. Jt Bone Spine.

[22] MVC N, Adrait A, Baillet A, Trocmé C, Gottenberg JE, Gaudin P. Identification of cartilage oligomeric matrix protein as biomarker predicting abatacept response in rheumatoid arthritis patients with insufficient response to a first anti-TNFα treatment. Jt. Bone Spine. 2019;86:401–3. 10.1016/j.jbspin.2018.09.005 30243783

[23] Nguyen MVC, Courtier A, Adrait A, Defendi F, Couté Y, Baillet A, et al. Fetuin-A and thyroxin binding globulin predict rituximab response in rheumatoid arthritis patients with insufficient response to anti-TNFα. Clin Rheumatol. 2020;39:2553–62. Available from: http://www.ncbi.nlm.nih.gov/pubmed/32212002. 10.1007/s10067-020-05030-6 32212002

[24] van Gestel AM, Prevoo ML, van’t Hof MA, van Rijswijk MH, van de Putte LB, van Riel PL (1996). Development and validation of the European League Against Rheumatism response criteria for rheumatoid arthritis. Comparison with the preliminary American College of Rheumatology and the World Health Organization/International League Against Rheumatism Cri. Arthritis Rheum.

[25] Smolen JS, Landewé RBM, Bijlsma JWJ, Burmester GR, Dougados M, Kerschbaumer A, et al. EULAR recommendations for the management of rheumatoid arthritis with synthetic and biological disease-modifying antirheumatic drugs: 2019 update. Ann Rheum Dis. 2020;79:685–99. annrheumdis-2019-216655. Available from: http://ard.bmj.com/lookup/doi/10.1136/annrheumdis-2019-216655. 10.1136/annrheumdis-2019-216655 31969328

[26] Martínez C, Ortiz AM, Juarranz Y, Lamana A, Seoane IV, Leceta J (2014). Serum levels of vasoactive intestinal peptide as a prognostic marker in early arthritis. PLoS One.

[27] Abasolo L, Ivorra-Cortes J, Leon L, Jover JA, Fernández-Gutiérrez B, Rodriguez-Rodriguez L. Contribution of the bone and cartilage/soft tissue components of the joint damage to the level of disability in rheumatoid arthritis patients: a longitudinal study. Clin Rheumatol. 2019;38:691–700. Available from: http://www.ncbi.nlm.nih.gov/pubmed/30328025. 10.1007/s10067-018-4335-4 30328025

[28] Pan W (2001). Akaike’s information criterion in generalized estimating equations. Biometrics.

[29] Leon L, Abasolo L, Fernandez-Gutierrez B, Jover JA, Hernandez-Garcia C. Direct medical costs and their predictors in the EMAR-II cohort: “variability in the management of rheumatoid arthritis and spondyloarthritis in Spain”. Reumatol Clin. 2018;14:4–8. Available from: http://www.ncbi.nlm.nih.gov/pubmed/27810462. 10.1016/j.reuma.2016.09.006 27810462

[30] Manca A, Hawkins N, Sculpher MJ (2005). Estimating mean QALYs in trial-based cost-effectiveness analysis: the importance of controlling for baseline utility. Health Econ.

[31] Little RJ, D’Agostino R, Cohen ML, Dickersin K, Emerson SS, Farrar JT, et al. The prevention and treatment of missing data in clinical trials. N. Engl. J. Med. 2012;367:1355–60. 10.1056/NEJMsr1203730 PMC3771340 23034025

[32] Leon L, Rodriguez-Rodriguez L, Rosales Z, Gomez A, Lamas JR, Pato E (2016). Long-term drug survival of biological agents in patients with rheumatoid arthritis in clinical practice. Scand J Rheumatol.

